# Proteomic characterization of HIV-modulated membrane receptors, kinases and signaling proteins involved in novel angiogenic pathways

**DOI:** 10.1186/1479-5876-7-75

**Published:** 2009-08-27

**Authors:** Suraiya Rasheed, Jasper S Yan, Adil Hussain, Bruce Lai

**Affiliations:** 1 Laboratory of Viral Oncology and Proteomics Research Department of Pathology, Keck School of Medicine, University of Southern California, 1840 N Soto St, Los Angeles, CA 90032-3626, USA

## Abstract

**Background:**

Kaposi's sarcoma (KS), hemangioma, and other angioproliferative diseases are highly prevalent in HIV-infected individuals. While KS is etiologically linked to the human herpesvirus-8 (HHV8) infection, HIV-patients without HHV-8 and those infected with unrelated viruses also develop angiopathies. Further, HIV-Tat can activate protein-tyrosine-kinase (PTK-activity) of the vascular endothelial growth factor receptor involved in stimulating angiogenic processes. However, Tat by itself or HHV8-genes alone cannot induce angiogenesis *in vivo *unless specific proteins/enzymes are produced synchronously by different cell-types. We therefore tested a hypothesis that *chronic *HIV-*replication in non-endothelial cells *may produce novel factors that provoke angiogenic pathways.

**Methods:**

Genome-wide proteins from HIV-infected and uninfected T-lymphocytes were tested by subtractive proteomics analyses at various stages of virus and cell growth *in vitro *over a period of two years. Several thousand differentially regulated proteins were identified by mass spectrometry (MS) and >200 proteins were confirmed in multiple gels. Each protein was scrutinized extensively by protein-interaction-pathways, bioinformatics, and statistical analyses.

**Results:**

By functional categorization, 31 proteins were identified to be associated with various signaling events involved in angiogenesis. 88% proteins were located in the plasma membrane or extracellular matrix and >90% were found to be essential for regeneration, neovascularization and angiogenic processes during embryonic development.

**Conclusion:**

Chronic HIV-infection of T-cells produces membrane receptor-PTKs, serine-threonine kinases, growth factors, adhesion molecules and many diffusible signaling proteins that have not been previously reported in HIV-infected cells. Each protein has been associated with endothelial cell-growth, morphogenesis, sprouting, microvessel-formation and other biological processes involved in angiogenesis (p = 10^-4 ^to 10^-12^). Bioinformatics analyses suggest that overproduction of PTKs and other kinases in HIV-infected cells has *suppressed *VEGF/VEGFR-PTK expression and promoted *VEGFR-independent *pathways. This unique mechanism is similar to that observed in neovascularization and angiogenesis during embryogenesis. Validation of clinically relevant proteins by gene-silencing and translational studies *in vivo *would identify specific targets that can be used for early diagnosis of angiogenic disorders and future development of inhibitors of angiopathies. This is the first comprehensive study to demonstrate that HIV-infection alone, without any co-infection or treatment, can induce numerous "embryonic" proteins and kinases capable of generating novel *VEGF-independent *angiogenic pathways.

## Background

Angiogenesis, or the formation of new blood vessels from the existing ones, is an essential biological process for maintaining numerous physiological functions ranging from cell growth, proliferation, repair of damaged cells to wound-healing *in vivo *[1-3]. Throughout the life of an individual and during embryonic development, various pro-angiogenic and anti-angiogenic factors (i.e. promoters and inhibitors of angiogenesis respectively) produced by various cell types maintain a balance between neovascularization and angiogenesis programs in a cyclic manner [4,5]. Exactly how abnormal angiogenic signals are generated *in vivo *is not well-understood, but an imbalance in the production of one or more critical factors can alter the protein-protein interaction pathways and induce angiogenic anomalies including inflammation, vascular dementia, hemangioma, dysfunctional uterine bleeding, ovarian hyperstimulation and choroidal/intraocular disorders to name a few [1,6]. Angiogenesis is also critical for cancer metastasis, diabetic blindness, age-related macular degeneration, rheumatoid arthritis, psoriasis, and for the development of new blood vessels that supply oxygen and nutrients to the body when aortas are clogged (thrombosis) [2,6].

In both the neoplastic and non-neoplastic diseases, endothelial cells have been shown to express various isoforms of the vascular endothelial growth factors (VEGFs) which bind to their cognate VEGF receptors (VEGFRs), activate their associated protein tyrosine kinases (PTKs) and stimulate endothelial cell growth through angiogenic pathways [3,6,7]. However, endothelial cells can be activated by various cytokines, phosphorylated proteins and other factors that are essential not only for cell growth but also for maintaining an activated state of the stimulated endothelial cells [2,8]. In the absence of specific cytokines and diffusible signaling proteins, VEGF by itself is not sufficient to trigger expression of numerous enzymes and proteins required for the development of a network of blood vessels from the existing vasculature [8,9].

### Angiogenic Factors are also produced by Pathogenic Viruses

Etiologic factors involved in different types of vasculopathies in humans have not been fully explored. However, in the *absence of any tumor growth *many DNA or RNA viruses have been shown to cause vascular lesions *in vivo *or produce proangiogenic factors *in vitro*. For example, the human herpes simplex virus type 1 (HSV-1)-infected ocular cells produce IL-6, which stimulates *uninfected, avascular *corneal cells to secrete VEGF and provoke neovascularization in the eye [10]. Infection with the Epstein-Barr virus (EBV) enhances production of many cytokines and causes angiogenic cutaneous tumors [11]. The dengue virus, causes hemorrhagic fever and vascular lesions in humans, produces interleukin-4 (IL-4), IL-8, IL-6, IL-10, GM- colony stimulating factor (CSF), interferon-gamma (INF-gamma) and tumor necrosis factor alpha (TNF-alpha) [12]. The human parapoxvirus causes extensive skin vasculopathies and the pseudocowpox viral genome induces *viral gene-encoded *VEGF homologues (i.e. VEGF-like factors) [13,14]. Likewise, the common human rhinovirus infection produces factors that promote angiogenesis in bronchial epithelial cells [15].

One of the best-studied models of angiogenesis is Kaposi's sarcoma (KS), a highly vascular tumor that is rare in the general population but occurs frequently in human immunodeficiency virus (HIV)-infected individuals [16-18]. However, KS is etiologically associated with the human herpesvirus-type-8 (HHV-8) infection since HHV8-genome itself encodes a viral G-protein-coupled receptor (vGPCR), which activates both oncogenic and angiogenic pathways in the *presence or absence of HIV-coinfection *[17,19,20].

Many HIV-infected patients, who may or may not be infected with HHV8, develop intraepithelial neoplasia, hemangiomas, lymphomas, angiosarcomas, myelodysplastic angiogenic syndrome and other angiopathies [21-23]. The HIV-encoded transcriptional transactivator (Tat) protein has been implicated in angiogenesis because it binds VEGFR and stimulates endothelial cell growth [17]. However, its binding-affinity is not as strong as that of the natural *cellular *VEGFs and the avidity of Tat interaction with VEGFR is *dependent on specific *cytokines produced locally by endothelial cells, cancer cells or other virus-infected and uninfected cell types *in vivo *[10,13,24,25]. Further, the *activated state of endothelial cells must be maintained continuously *during the numerous biological processes that lead to angiogenesis. These data suggest that while Tat *synergizes *the effects of many viral and cellular factors during the complex biological processes of angiogenesis, Tat alone or individual cytokines by themselves do not induce angiogenesis in mice.

The molecular mechanisms involved in HIV-induced vasculopathies in humans are difficult, if not impossible to study because most patients are co-infected with different pathogenic viruses such as HSV-1, HSV11, EBV, hepatitis B virus (HBV), hepatitis C virus (HCV), human papilloma virus (HPV) and different bacterial and fungal microorganisms. Consequently, cellular changes induced by HIV alone *in vivo *can not be distinguished from those produced by other viruses or pathogenic organisms co-inhabiting the same individual, unless separate protein profiles of each class of different infectious agents are established first. We therefore tested a hypothesis that *chronic *HIV-*replication in non-endothelial cells *induces novel cellular proteins that provoke specific protein-protein interactions along the angiogenic pathways. Although most *in vitro *studies have utilized endothelial cells derived from early KS lesions or human veins (by necessity), in this study we preferred to use T-cells because some differentiated endothelial cells may already produce proangiogenic cytokines in response to changes in the cellular milieu or alternatively, factors that are essential for endothelial cell activation may be experimentally induced [26,27]. Herein, we report that HIV- infected human T-cells produce numerous kinases, adhesion molecules and other angiogenic factors (not encoded by HIV-genome) that are capable of initiating and promoting novel VEGF-*independent *pathways. These mechanisms are similar to those observed during embryonic development, neovascularization and angiogenesis.

## Experimental design and methods

To identify possible factors that can be associated with HIV-infection alone, we used a single-cell-cloned human T-cell line (RH9) consisting of a homogeneous population of cells [28]. These cells are highly susceptible to the replication of most global HIV-strains tested including those that are preferentially "macrophage/monocyte-tropic" (SR personal observation). The RH9 cells do not induce cytopathic effects but occasionally, when some chronically infected cultures exhibit syncytia, uninfected counterpart cells are added to maintain long-term HIV-infected cell lines.

The choice of T-cells for HIV infection was also based on the fact that T-cells, together with monocytes and macrophages present at the portal of entry *in vivo *are the first cell types to be infected soon after HIV-exposure. Our experiments were deliberately designed to avoid the use of primary T-cells for HIV-infection due to the genetic heterogeneity and sample-to-sample variation in the susceptibility of freshly cultured human peripheral blood mononuclear cells (PBMC) (SR unpublished data). Since HIV-infected individuals harbor a variety of different strains (present as quasispecies *in vivo*), we used a biologically cloned HIV strain (X4) in order to have better reproducibility and consistency of results from experiment to experiment. This methodology reduced variations in their replication potentials.

While several HIV-infected T-cell lines or Tat-transfected T-cell lines have been used to study HIV-infected proteomes and gene expression profiles, all of these analyses were conducted after a short time (24–48 hrs) of infection or transfection of cells [29-32]. Given that most HIV-diseases including vasculopathies are developed after several years of chronic infection, we compared genome-wide proteins from HIV-infected and counterpart uninfected T-lymphocytes over a period of two years by subtractive proteomics, bioinformatics and statistical analyses. These studies were designed to evaluate *only *the differentially regulated (i.e. upregulated, downregulated or *de novo *induced proteins post-HIV infection), and *not *the entire proteome of the HIV-infected or uninfected cells. Finally, all experiments were conducted in the *absence *of other pathogenic viruses or microbes that may produce proangiogenic factors.

### Virus Infection for Proteomics Studies

Approximately 10^9 ^cells were plated in each of the two large flasks at a density of 2 × 10^6 ^cells per ml in RPMI 1640 medium supplemented with 20% fetal bovine serum (FBS), 2 mM glutamine and 2 μg/ml polybrene. After 16–18 hours (h), one culture was infected with HIV at a multiplicity of infection of one (MOI = 1) and both infected and uninfected cultures were incubated at 37°C in an atmosphere of 5% CO_2_. After 1.5 h, all cells from both flasks were harvested separately, washed with phosphate buffered saline (PBS) and transferred to *new *flasks with fresh medium without polybrene.

Numerous experiments were conducted over a period of more than two years and changes in protein profiles were analyzed in relation to various HIV-associated dysfunctions/diseases. One experiment was conducted for approximately 3 months and duplicate samples from HIV-infected and counterpart uninfected samples were tested at 14 time points by proteomics analyses. These samples ranged from 1.5 h to 96 days (d) post-infection (3 h, 6 h, 12 h, 24 h, 48 h, 4 d, 10 d, 14 d, 20 d, 26 d, 28 d, 47 d and 96 d). In subsequent experiments, samples were harvested at the peak of HIV-replication (i.e. from 10 to 26 days). Given that most HIV-associated diseases develop after a chronic infection, we tested an additional ten different chronically HIV-infected and uninfected counterpart cells selected randomly over a period of two years i.e. at various stages of virus replication and cell growth. This large sample size was necessary in order to select highly reproducible protein spots in multiple gels and for testing many quality-control samples used for standardization of experiments such as lyophilized E. coli extract, commercially available purified proteins and a single extract of HIV-infected and uninfected cells.

### Isolation of Plasma Membrane and Extracellular Matrix Proteins

A major goal of this study was to identify cell surface proteins involved in generating HIV-modulated signals that disrupt normal cellular functions and drive infected cells in specific directions. Over the years our laboratory has developed a rapid sequential extraction procedure to successfully isolate functionally relevant and naturally occurring plasma membrane and extracellular matrix proteins [33,34]. All proteins were isolated by unbiased approaches (i.e. without the use of special ligands, antibodies or ion exchange columns or liquid chromatography for capturing or purifying specific proteins). Although this may not be an ideal method for identifying the entire proteome, this method was excellent for identifying many differentially expressed signal transduction molecules. Briefly, aliquots of 10^7 ^cells from each of the HIV- infected and uninfected cultures were removed at various time points as indicated above, and washed with PBS by low speed centrifugation twice and once with normal saline (0.9% NaCl). The cell pellets were lysed rapidly for 15 seconds using (8 M Urea, 2% (w/v) CHAPS, 2% mercaptoethanol, 2.5% protease inhibitor cocktail, and 150 units/200 μl endonuclease). Each lysate was then vortexed gently and sonicated for 2 seconds followed by centrifugation at 14,000 rpm for 10 minutes. Just before loading the gels, the clarified supernatant from the lysate was centrifuged again at 100,000 × g for 90 minutes in a high-speed centrifuge and processed for protein fractionation by two-dimensional gel electrophoresis. All proteins were separated first by isoelectric focusing on various pH gradients (3 to10) and size fractionated in the second dimension by gel electrophoresis on gradient polyacrylamide gels (6–18%).

Electrophoretically separated proteins in the gels were washed 3× with double-distilled H_2_O and stained with Coomassie Brilliant Blue for 30 minutes and de-stained in 15% (v/v) methanol, 7% (v/v) acetic acid for a minimum of three hours. Several Coomassie-stained gels were counterstained with Sypro Ruby Red (SRR) fluorescent dye *after *the gels were scanned for image-analysis and double stained gels were scanned again. Since fluorescent signals of SRR are photostable and comparable to Cy3 and Cy5 dyes [35], this procedure enhanced the sensitivity of some light-colored spots and reduced non-specific spot identity.

### Bioinformatics and Statistical Analyses for Identification of Angiogenic Proteins

Genome-wide protein profiles of both the infected and uninfected counterpart cells were compared and evaluated by subtractive proteomics analyses overtime i.e. at different stages of virus and cell growth. *Only *those proteins that were clearly identified by Matrix Assisted Laser Desorption Ionization-Time of- Flight (MALDI-TOF) mass spectrometry (MS) in multiple gels were included in the final analyses. Further, any "new" proteins (i.e. hypothetical proteins) identified by MS or peptide fingerprinting with low Molecular Weight Search (MOWSE) Scores (p = 0.05 or more) in any gel were excluded from the current analyses regardless of the intensity of the stain.

All protein profiles from the HIV-infected and uninfected cells were compared and analyzed by a variety of subtractive computer-based approaches. Integrated programs for accuracy analyzed all proteins by calculating means and standard deviations for quantitative evaluations of proteins in both HIV-infected and uninfected controls. To identify HIV-modulated proteins related to angiogenesis, we have used several bioinformatics programs and gene/protein databases including the Online Mendelian Inheritance in Man (OMIM), a database of human genes and genetic disorders. The Ingenuity Pathway Analyses (IPA) Systems and Computational Biology programs were used to analyze global canonical and protein-interaction pathways for each of the identified proteins. Each protein was also functionally categorized to identify possible roles in the numerous stepwise processes, from HIV-induced cell activation to the formation of a network of new blood vessels from the existing endothelial cells.

Each differentially regulated protein was analyzed for its biological significance relative to those present in the global gene/protein databases available in the public domains and cell type-specific functionality by the use of Ingenuity-IPA/computational programs. The numbers of "focus" proteins (Table 1) were annotated in relation to the total number of genes/proteins known to be associated with various essential biological processes involved in endothelial cell growth, formation of blood vessel and other categories recorded in the Ingenuity's knowledgebase. The p-values were calculated using IPA and the right-tailed Fisher Exact Test for each of the various biological/cellular processes involved in angiogenesis. All p-values were less than 0.0001 (Table 1).

### Protein-Protein Interaction Pathway Analyses

The Ingenuity Pathway Analyses (IPA) Systems and the direct Interaction Function Bioinformatics Programs of Stratagene Pathway Architect 2.0.1 were used to analyze protein-protein interaction pathways. All dysregulated proteins were uploaded and function-specific pathways were generated automatically by using IPA as well as Stratagene Architect programs. Although similar pathways were constructed by the two programs, the protein-protein interaction pathways presented herein were made by the Stratagene Architect program.

## Results and discussion

Cell culture supernatants from all experimentally HIV-infected cells showed an exponential increase in the p24 antigen levels tested over time by the enzyme-linked immunoassays. Although many HIV-encoded proteins (gag-p24, Tat, Rev, Vpu, Vpr, Vif, gp120, gp41 and the polymerase) were identified by mass spectrometry (MS) in various protein-complexes, in this study we have focused on the identification of HIV-modulated *cellular *proteins *only *(i.e. not encoded by viral genes).

### Functional Categorization of Cellular Proteins

Comprehensive MS analyses of several thousand proteins confirmed more than 200 proteins from multiple gels run at different phases of cell growth and virus replication over time. Results presented herein have been consolidated from proteomics data generated over a period of > 2 years. Each of the differentially regulated proteins was functionally categorized by the use of bioinformatics programs that integrated biological information currently located in several global databases including Ingenuity Systems' knowledgebase of the Functional Repository of Human Genes. We have identified 31 proteins that have been deemed essential for numerous molecular functions involved in neovascularization (i.e. formation of blood vessels *de novo *in the embryo) or in angiogenesis (i.e. generation of new blood vessels from the existing vasculature). Full name, abbreviation and accession number for each protein are listed according to the information available on the latest Swiss-Prot/UniProt Public databases (Table 1). While a p-value of < 0.05 is generally considered significant for a specific function, each of the proteins included in this study was highly significant for multiple essential functions associated with angiogenesis (p = 10^-4 ^to 10^-12^) (Table 1).

**Table 1:** HIV-Modulated Proteins Associated With Essential Steps During Angiogenesis

**Protein names and Abbreviations**	**Accession #**	**P-Value related to Angiogenesis**
***1. Activation of T-Cells: Transcriptional and Translational Reprogramming***		
T-cell receptor zeta chain, tyrosine-protein kinase (ZAP-70)	P43403	4 × 10^-5^
TNF receptor (TNR) superfamily # 9 (TNR9)	Q07011	8 × 10^-8^
Complement receptor 3 (CO3/C3)*	P01024	1 × 10^-6^
Beta type serine/threonine protein kinase C (PKC)*	P05771	8 × 10^-12^
***2. Regulation of Cell Cycle: Lipid Kinase, Endothelial zinc finger and p53-binding protein***		
Phosphatidylinositol-4-phosphate3-kinase C2-beta (P3C2B/PI3K)	O00750	8 × 10^-12^
Endothelial zinc finger protein (ZNF71)	Q9NQZ8	N/A
Tumor suppressor p53-binding protein 1 (TP53B)	Q12888	2 × 10^-8^
***3. Augmentation of Cell Growth: Overexpression of Receptor Protein Tyrosine Kinases***		
ERBB2 receptor protein tyrosine kinase (ERB2)	P04626	2 × 10^-10^
Growth factor receptor-bound protein 2 (GRB2)	P62993	8 × 10^-12^
Vascular endothelial cell growth factor C (VEGFC) **Not expressed**	P49767	2 × 10^-10^
VEGF receptor tyrosine kinase VEGFR-2 (VGFR2) **Not expressed**	P35968	8 × 10^-12^
***4. Survival of Newly Formed Cells: Serine-Threonine Protein Kinase C (PKC) and Adapter Proteins***		
Beta type serine/threonine protein kinase C (PKC)*	P05771	8 × 10^-12^
Protein kinase C-binding protein NELL1 (NELL1)	Q92832	8 × 10^-8^
Annexin VI (ANXA6)	P08133	3 × 10^-4^
14-3-3 protein gamma (143G)	P61981	3 × 10^-10^
***5. Mitogenic Signaling Cascade; Mitogen-activated Protein Kinase***		
Mitogen-activated protein kinase (MAPK3)	P27361	8 × 10^-12^
CRK-like adapter protein (CRKL)	P46109	5 × 10^-10^
***6. Balanced Cell Growth or Adhesion: Anti-angiogenic G-Protein Coupled Receptors***		
Brain-specific angiogenesis inhibitor 1 (BAI1)	O14514	2 × 10^-10^
Brain-specific angiogenesis inhibitor 3 (BAI3)	O60242	N/A
***7. Adhesion, Differentiation & Cell Migration: Focal Adhesion Kinase, Adhesion Receptor & Enzymes***		
Focal adhesion tryosine kinase 2 beta (FAK2)	Q14289	2 × 10^-9^
Alpha (V) beta (5) integrin (ITB5)	P18084	2 × 10^-9^
Nitric-oxide synthase (NS2A)	P35228	2 × 10^-9^
Fibronectin Precursor (FINC)	P02751	1.5 × 10^-3^
Low molecular weight phosphotyrosine protein phosphatase (PPAC)	P24666	2 × 10^-9^
***8. Morphogenesis and Cell Migration: Laminins and other Cell Adhesion Molecules***		
Laminin beta-2 chain precursor (LAMB2)**Upregulated**	P55268	2 × 10^-8^
Laminin alpha-5 chain protein precursor (LAMA5)	O15230	2 × 10^-10^
Cadherin EGF LAG seven-pass G-type receptor 1 (CLR1/CELSR1)	Q9NYQ6	N/A
Protocadherin focal adhesion targeting (FAT2)	Q9NYQ8	7 × 10^-6^
Golgi apparatus Protein 1 (GLG1)	Q92896	N/A
***9. Cell Permeability & Sprouting: Myosin Light Chain Kinase, Aggrecans & Peptidase***		
Myosin light chain kinase smooth muscle/non-muscle isoezymes (KMLS)	Q15746	3 × 10^-12^
ADAMTS-9 (ATS9)	Q9P2N4	4 × 10^-4^
Complement receptor 3 (CO3/C3)*	P01024	1 × 10^-6^
***10. Preservation of Differentiated Cellular Phenotype: Coagulation-related Factor***		
Von Wilebrand factor (VWF)	P04275	2 × 10^-7^

**Figure 1:**
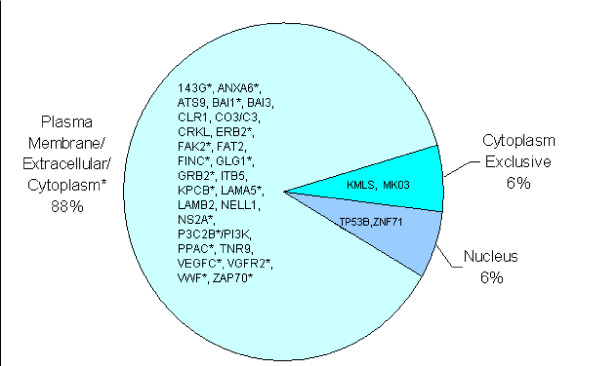
**Cellular Locations of Differentially regulated Proteins in HIV- Infected T-Cells**. The pie-chart illustrates cellular localization of 31 proteins that were upregulated, downregulated or induced *de novo *post-HIV infection. Protein abbreviations are according to the Swiss-Prot/Uni-Prot knowledgebase. Asterisks (*) represent proteins that have been primarily localized in the plasma membrane or extracellular matrix but have been occasionally reported to be expressed in the cytoplasm or other locations. The cytoplasmic proteins include KMLS and MAPK3 (MKO3) and nuclear proteins are TP53B and ZNF71. Full protein names, abbreviations and accession #s for each of all proteins are provided in Table 1.

**Figure 2:**
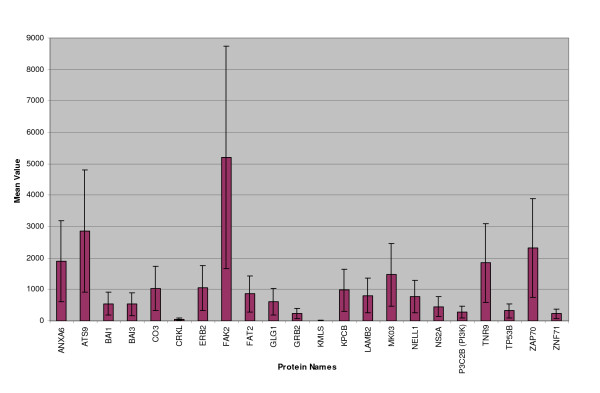
**Proteins Detected Exclusively in HIV-Infected Cells**. Graph showing proteins that were detected exclusively in HIV-infected cells (i.e. these proteins were not detected in counterpart uninfected cells at any time during the study). Although integrin (ITB5) was expressed in HIV-infected cells only, the small quantities could not be charted on the scale used. X-axis shows protein abbreviations according to Swiss-PROT/UniProt databases. Y-axis illustrates average of normalized quantity of specific protein spot computed automatically by the use of PDQuest program from multiple gels. Error bars represent one standard deviation of the range for each protein data. Full protein names, abbreviations and accession #s of each protein are provided in Table 1.

**Figure 3:**
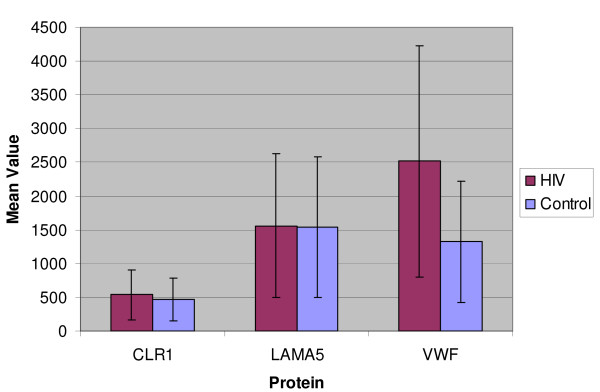
**Proteins slightly Upregulated or same values Post-HIV-infection**. Graphic representation of two proteins (LAMA5 and CLR1/CELSR1) showing approximately the same values as control post-HIV-infection. VWF was slightly upregulated following HIV infection but was not statistically significant in quantity. FINC could not be charted because of low levels. X-axis = protein abbreviations are from Swiss-PROT/UniProt. Y-axis = average of normalized quantities of proteins detected in multiple gels. Error bars represent one standard deviation for the range of each protein data. Full protein names and accession #s of each protein are provided in Table 1.

**Figure 4:**
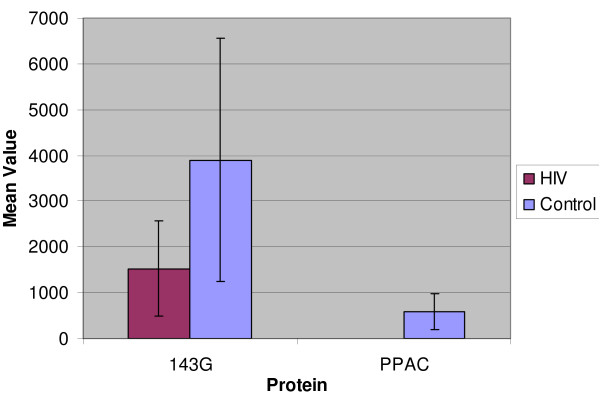
**Proteins Down-regulated post-HIV- infection**. Two proteins were downregulated (1433G and PPAC) post-HIV-infection of T-cells. X-axis = protein abbreviations according to SwissPROT). Y-axis = average of normalized quantities of the same protein detected in multiple gels. Error bars represent one standard deviation for the range of each protein data. Full protein names and accession #s of each protein are provided in Table 1.

HIV-modulated proteins significantly associated with essential biological steps in neovascularization and angiogenesis. Four proteins were upregulated, two were downregulated and all the rest (n = 25), were expressed *de novo *post-HIV-infection (i.e. not expressed in uninfected counterpart cells; Figures 1–4).

Since most of the proteins expressed in HIV-infected cells are multifunctional, the categorization of these proteins is only to facilitate a better understanding of numerous complex biological processes involved in angiogenesis. Thus, PKC is listed in categories #1 and #4 and C3/C03 is listed in #1 and #9

Approximately 88% (27 of 31) of the HIV-modulated proteins could be located to the plasma membrane or extracellular matrix of the infected cells (Figure 1). Functional categorization of the identified proteins indicated that each protein belonged to specific families of signal transduction molecules including receptor or non-receptor tyrosine kinases (ERBB2, ZAP70, FAK2), serine-threonine kinases (KMLS, MAPK3 and PKC), lipid kinase (P3C2B/PI3K), G-protein coupled receptors (BAI1, BAI3 and CLR1), adhesion molecules/cytoskeletal proteins (LAMA5, LAMB2, ITB5, FAT2, FINC), kinase adapters or binding proteins (GRB2, CRKL and NELL1), protease/peptidase (ATS9 and C3/CO3), regulatory enzyme (NS2A), integral membrane proteins (TNR9 and GLG1), calcium-binding protein (ANX-A6) and coagulation factor (VWF) (Figures 2 &3). Although numerous transcription factors were induced *de novo *or upregulated post-HIV-Infection of T-cells, in the present analysis, we have considered the endothelial cell-specific zinc finger transcription factor (ZNF71) induced by TNF alpha and (TP53B), as important regulatory proteins that may be necessary for the expression of cell-cycle genes/proteins during the complex biological processes of angiogenesis *in vivo*.

The VEGFR2 receptor and its growth factor ligand VEGFC were downregulated in HIV-infected cells, although detected only *once *in one of numerous acutely HIV-infected cultures tested. The PKC-regulatory protein 143G was expressed at a lower level in HIV infected cells compared to the uninfected controls. The quantities of LAMA5 and CLR1 were not much different between the infected and uninfected cells (Figure 3). In addition a phosphatase (PPAC) was completely suppressed after HIV-infection (i.e. detected only in the *uninfected *counterpart cells) (Figure 4). The downregulation of PPAC is considered to be significant because its *absence *is essential for *maintaining phosphorylation *of various tyrosine kinases and activation of endothelial cell growth *in vivo *[36].

The biological significance of all 31 proteins identified in this study was computed in relation to protein-interaction networks involved in angiogenesis (p = 8 × 10^-12^). This, we believe, is the first step toward developing a better insight into the molecular mechanisms by which pathogenic viruses such as HIV may initiate and/or promote angiogenesis in the infected host.

### Stepwise Analyses of Essential Biological Processes in Angiogenesis

Angiogenesis is a multifactorial biological process involving numerous steps including endothelial cell activation, degradation of basement membrane, cell proliferation, invasion, morphogenesis, sprouting, migration and stabilization of microvessel formation. Each step involves a series of extremely complex but well-orchestrated protein-protein interactions along various signaling pathways. To understand the biological significance of each protein, we have divided all proteins into 10 well-recognized biological events during neovascularization or angiogenesis (Table 1), and discussed putative functions of each protein in that category. Since most proteins are multifunctional, some overlap in the protein functions was inevitable.

#### Step 1- Activation of T-Cells: Transcriptional and Translational Reprogramming

As soon as the HIV envelope glycoproteins (gp120/gp41) bind to the T-cell receptor and co-receptors (CD4, CXCR4 and others), the cell surface proteins are clustered. This generates a cascade of signals from the plasma membrane to the cytoplasm and nucleus. As the new proteins are expressed, the HIV-infected cells are activated and are driven toward apoptotic pathways [37,38]. However, most activated cells also produce numerous cytokines, enzymes and other signal transduction molecules that invoke innate cellular immunity (to combat virus-infection) and may be critical for the survival of the infected cells. These proteins maintain cellular integrity during various phases of HIV replication and cell growth. Many proteins that are upregulated, downregulated or induced *de novo *post-HIV infection may also be necessary to compensate for the loss or disruption of essential physiological functions performed by the T-lymphocytes *prior *to HIV infection.

Among a diverse family of multifunctional signaling proteins induced *de novo *in HIV-infected cells, the protein tyrosine kinases, the serine/threonine kinases and many regulatory enzymes appear to play major roles in T-cell activation and global reprogramming of the transcriptional and translational activities that lead to novel interaction pathways (Table 1).

##### Zeta Chain Tyrosine-Protein Kinase (ZAP-70)

**Figure 5:**
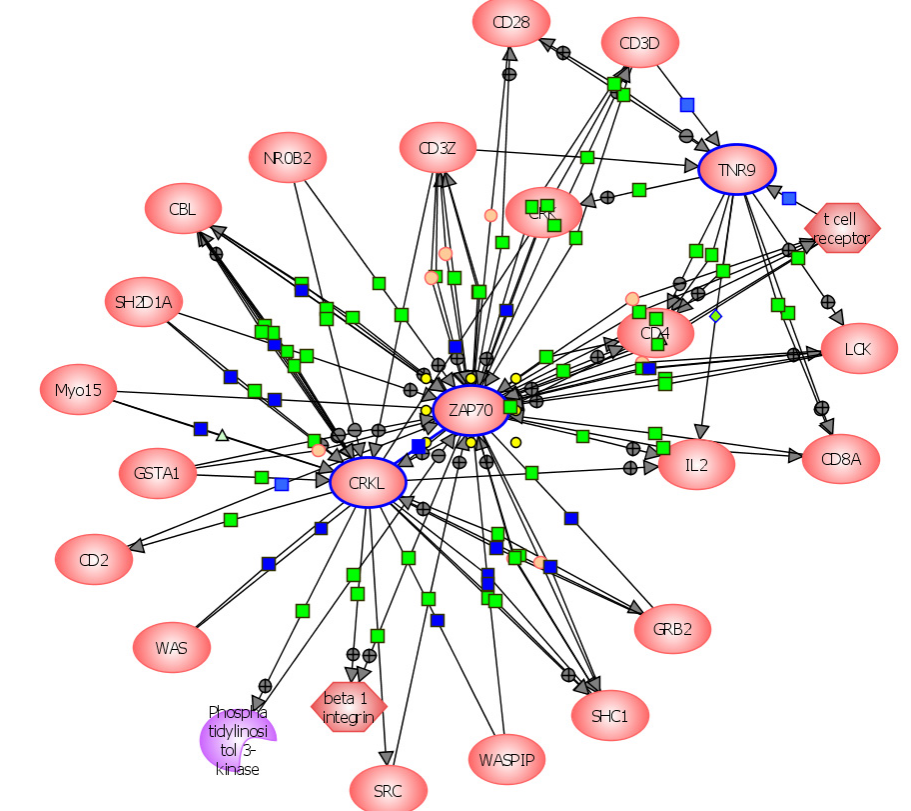
**T-Cell Activation Pathways Generated by HIV-Modulated Proteins**. Graphic representation of major proteins and kinases involved in T-cell activation; the pathways were constructed by the direct Interaction Function Bioinformatics Programs of Stratagene Pathway Architect 2.0.1. All proteins were uploaded and function-specific pathways were generated automatically; blue outlines around red ovals (ZAP 70, CRKL, and TNR9), indicate the activated proteins. Note numerous cell surface proteins including PI3K involved in T-cell activation pathways. Lines between red ovals denote major interactions; green circles represent small molecule interactions. Full names of all protein abbreviations and accession numbers are listed in Table 1.

The zeta chain protein tyrosine kinase (ZAP70-PTK) was expressed exclusively in HIV-infected cells (Table 1; Figure 2). This kinase is associated with the zeta chain of the T-cell receptor (TCR) expressed on the plasma membrane. The tyrosine kinase activity of this receptor phosphorylates multiple tyrosine residues of many functionally important proteins (Figure 5) [39,40].

An important function of ZAP70 protein kinase in HIV infected T-lymphocytes appears to be the suppression of CD4-mediated CD3 signaling which selectively impairs T-cell functions, reduces immune responses, induces anergy and stimulates apoptosis in T-cells of both HIV-infected and uninfected individuals [39] (p = 5 × 10^-8^). However, in promonocytic cells, the HIV-encoded Nef protein activates the Src/Syk protein tyrosine kinase (SKF) activity and recruits ZAP-70 [41]. These multi-kinase complexes have been reported to induce a cascade of signals which cause downregulation of major histocompatibility complex-1(MHC-I) via a membrane associated lipid kinase, phosphatidylinositol-4-phosphate3-kinase C2-beta (PI3K) pathway (Figures 2, 5), [41,42]. Although this interaction also affects immune evasion of HIV-infected CD4+ T-cells, our experimentally-infected cells expressed PI3K, concomitantly with the activation of ZAP-70 and other protein tyrosine kinases. Co-expression of these proteins is critical for efficient coupling and antigen recognition of several intracellular signal transduction molecules and may also promote cell-to-cell contacts and increased HIV-spread [40,43].

An interesting finding relevant to our study was that the upregulation of ZAP-70 PTK correlates *negatively *with the expression of VEGF in patients with highly malignant, *angiogenic *chronic B lymphocytic leukemia (CLL) [44,45]. Although B-cell functions are not compromised by an increase in ZAP70 kinase, its expression on the surface of CLL cells has been linked to the increased angiogenesis and poor prognosis of this cancer [45,46]. On the contrary, *absence *of ZAP-70 expression was a good prognosticator for CLL (i.e. with less or no angiogenesis) although VEGF was expressed [44]. These data suggest that VEGF-independent pathways were involved in CLL malignancy. Our proteomics and bioinformatics analyses of HIV-infected cells are consistent with these findings since expression of ZAP-70 PTK and other PTK-containing proteins was associated with concomitant downregulation of both the VEGF and its cognate receptor VEGFR (p = 2.6 × 10^-3^).

##### Tumor Necrosis Factor Receptor (TNR9)

One of the most frequently expressed cytokines during HIV-infection *in vitro *or *in vivo *is the tumor necrosis factor (TNF). The receptor for TNF belongs to the superfamily # 9 (TNR9) (synonyms: 4-1BB ligand receptor or CD137 antigen) was expressed *de novo *in the experimentally HIV-infected cells (Table 1, Figure 2). This receptor is important for the survival and maintenance of functional changes in the CD4 and CD8 cells as immune effectors (p = 8 × 10^-8^), [47].

The TNR9 receptor belongs to the TNF-nerve growth factor (NGF) receptor family and is activated by TNF or related factors that are produced by most virus-infected cells [48]. Expression of TNR9 receptor facilitates clustering of T-cell receptors at the cell surface of HIV-infected cells. This interaction is conducive to activation of protein kinases, and nuclear factor kappa B-associated signal transduction pathways involved in the regulation of cell growth, differentiation and inflammatory processes that precede angiogenesis (p = 7 × 10^-4^), (Figure 5), [47-50].

Expression of TNR9 is also linked to the activation of HIV-1 replication from latently infected CD4+ T cells [50,51]. Upregulation of this receptor in HIV-infected cells may therefore be essential for the sustained T-cell stimulation and production of novel proteins that are needed to facilitate virus replication and synthesize virus particles without killing the cell. Although the expression of TNF has been reported in many viral and microbial infections, the upregulation of this factor in cancer cells has been associated with the induction of angiogenic factors [52].

##### Complement Receptor 3 (CO3/C3)

The complement receptor 3 (CO3/C3) was detected only in HIV-infected cells (Table 1; Figure 2). This protein is the first responder of the innate immunity and is critical for the protection of virus-infected hosts/cells. Since amino acid sequences of human C3 are similar to those of HIV- gp120 and gp41 envelope proteins, C3 can bind efficiently to different sites on the surface of T-cells and activate them [53,54]. Expression of C3 in HIV-infected cells increases the spread of virus to other cell types such as dendritic cells present in the peripheral blood of HIV-infected individuals [55-57].

One of the many critical functions of the C3 (and C5 peptidases) is to stimulate chemotaxis and eventually contribute to the development of choroidal neovascularization [58,59]. These proteins also enhance permeability of vasculature and cell migration during embryogenesis (p = 4 × 10^-4^). Bioinformatics analyses indicates that a coordinated expression of ZAP70, TNFR9 and C3, as well as the release of these proteins in the blood of HIV-infected individuals, may be significantly involved in the initial growth and expansion of endothelial cells in early phases of angiogenesis (p = 7 × 10^-4^).

##### Protein Kinase C Beta Type (PKC)

Protein kinase C beta type (PKC) is a multifunctional kinase, expressed exclusively in the HIV infected cells (Table 1; Figure 2). This kinase is essential for a wide range of cellular functions including survival of activated T-cells (i.e. protection of HIV-infected cells from apoptosis), cell growth, and angiogenesis. Presence of PKC induces many intracellular signaling molecules that are not only critical for the completion of virus life cycle [60,61], but are also associated with T-cell activation and hyporesponsiveness of these cells [62].

#### Step 2- Cell Cycle Regulation: Lipid Kinase, Endothelial Zinc Finger, p53-binding protein

##### Phosphatidylinositol-4-Phosphate3-Kinase C2-beta (PI3K) Lipid Kinase

One of the first sets of signals generated in response to extracellular stimuli involves the membrane-associated lipid kinase phosphatidylinositol-4-phosphate3-kinase C2-beta (PI3K or P3C2B). This kinase was induced *de novo *in HIV-infected T-cells (Table 1; Figure 2) and is considered essential for the activation of these cells. The PI3K preferentially phosphorylates phosphoinositide substrates that are necessary for cell cycle-related activities, DNA repair and cell proliferation [63,64].

The expression of PI3K is necessary for many physiological functions but the production of this lipid kinase may be enhanced by a variety of newly induced cytokines and the HIV-encoded Tat protein expressed in the HIV-infected cells [64,65]. Co-expression of PI3K with other kinases discovered in this study may also be necessary for cell survival (i.e. to keep the apoptotic pathways suppressed) in the HIV-infected T-cells and maintenance of the overall health and metabolism of activated cells during virus replication.

Our bioinformatics analyses indicate that a coordinated expression of PI3K with protein tyrosine kinases, serine-threonine kinases and other signaling proteins in our experimentally HIV-infected cells is critical for the controlled growth of newly made endothelial cells. Thus, concomitant expression of cell cycle genes, PI3K, MAPK and FAK2 together with interacting partners ERBB2, GRB2 and integrin v-beta (ITB5) in the HIV-infected T-cells is central to the endothelial cell proliferation which is directly relevant to various biological processes involved in angiogenesis. PI3K is also recruited by a phosphotyrosine signaling complex containing the activated receptor such as ERBB2 and a tyrosine kinase associated adapter protein GRB2 [66]. Another important function of PI3K is its regulatory role in the formation of tubular structures (vessels) during angiogenesis [67], through a well-coordinated expression of ITB5 and cell adhesion molecules that are crucial for endothelial cell motility and intracellular signaling pathways (p = 2 × 10^-5^).

##### Endothelial Cell-Specific Transcription Factor, Zinc Finger (ZF71)

Although numerous transcription factors were upregulated exclusively in our experimentally HIV-infected cells, the activation of endothelial cell-specific zinc finger ZF71 (synonym: EZFIT) in T-cells is noteworthy (Table 1; Figure 2). This transcription factor mediates a wide range of cellular functions such as transcriptional controls that regulate endothelial cell proliferation [68]. The ZF71/EZFIT mRNA levels were significantly upregulated when human umbilical vein cells were treated with TNF-alpha [68]. Our bioinformatics analysis suggests that the upregulation of TNR9, the receptor for TNF-alpha, and related factors in HIV-infected T-cells may have enhanced the expression of ZF71. Since TNF-alpha induces angiogenic factors in cancer cells [52] and upregulates production of signal transduction molecules including chemokines [69], it is probable that ZF71 promotes angiogenesis via the expression of tyrosine kinases and other critical enzymes in HIV-infected cells.

##### Tumor Suppressor p53-Binding Protein 1 (TP53B)

The tumor suppressor p53-binding protein 1 (TP53B or 53BP1) [70], was upregulated exclusively in HIV-infected T-cells (Table 1; Figure 2). This is a highly conserved nuclear protein associated with kinetochores (microtubule attachment points associated with centromere) and in some cells it shuttles between nucleus and cytoplasm [71]. Activation of this protein controls both the S phase and G2/M phase checkpoint controls (p = 2.6 × 10^-3^). Since TP53B also stimulates many different pathways immediately after the double stranded DNA is perturbed or damaged [71], it is likely that the integration of HIV provirus in the cellular DNA may have triggered the expression of cell-cycle-related pathways through TP53B.

Our bioinformatics and statistical analyses indicate that activation of TP53B concomitantly with numerous upregulated transcription factors, growth factors and enzymes in HIV-infected cells, may be significantly associated with cell survival and growth (p = 2 × 10^-4^). Further, co-expression of TP53B with the tyrosine kinase ERBB2, adhesion molecules, LAMB2 and LAMA5, is also significantly involved with the formation of vessels during embryonic development (p = 1.4 × 10^-3^).

#### Step 3- Augmentation of Cell Growth: Overexpression of Protein Tyrosine Kinases

##### The ERBB2 Receptor Protein Tyrosine Kinase

One of the most critical proteins induced by HIV appears to be the ERBB2 receptor protein tyrosine kinase (ERBB2-PTK; also known as HER-2/Neu or ERB2) (Table 1; Figure 2). The ERBB2 protein was originally isolated as a viral oncoprotein, which belongs to the epidermal growth factor (EGF) receptor family [72]. This protein was not detected in any of the numerous aliquots of the uninfected T-cells tested at different stages of cell growth, over a period of two years. Like most HIV-modulated proteins identified in the present study, expression of ERBB2 receptor has not been reported previously in HIV-infected cells.

**Figure 6:**
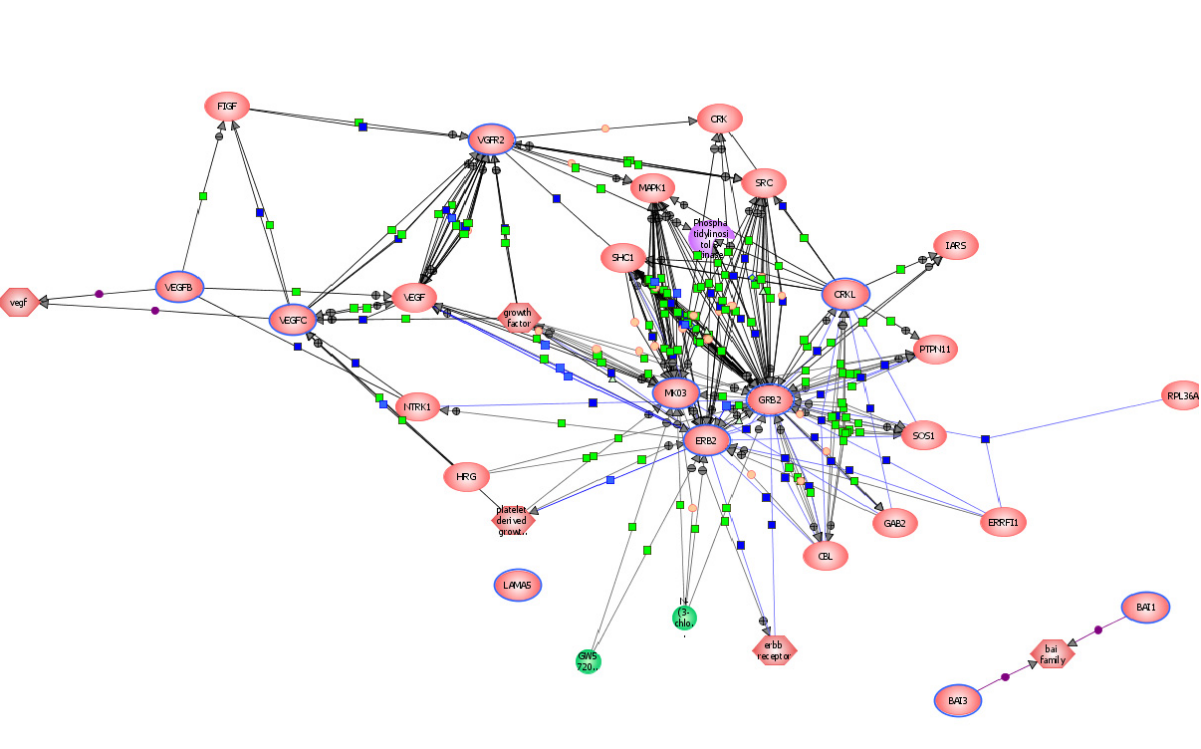
**Protein Interaction Pathways Involved in Augmentation of Cell Growth**. Cell growth-specific pathways were constructed by the direct Interaction Function Bioinformatics Programs of Stratagene Pathway Architect. All proteins were uploaded and function-specific pathways were generated automatically. Protein-protein- interactions involved in augmentation of cell growth and angiogenesis along VEGF-*independent *pathways. Note the VEGF-VEGFR interactions away from the ERBB2-GRB2-MAPK3 (MKO3). Most of the regulatory proteins and kinases discovered in these pathways are normally expressed during embryonic development. Full names of all protein abbreviations and accession numbers are listed in Table 1.

**Figure 7:**
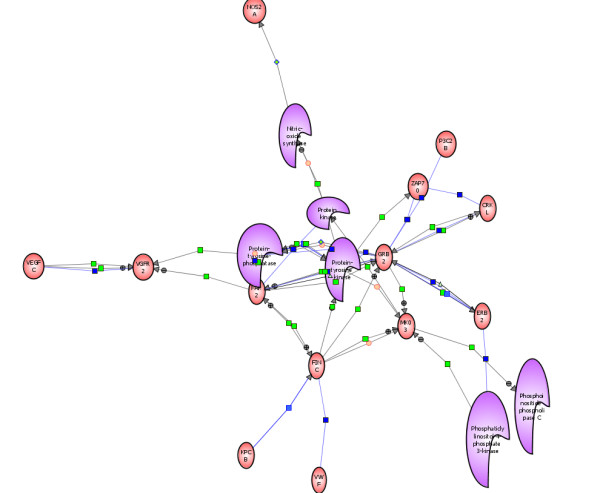
**Protein Tyrosine Kinase and other Major Kinases involved in Angiogenic Pathways**. Pathways were constructed by the direct Interaction Function Bioinformatics Programs of Stratagene Pathway Architect. ALL proteins mapped in this figure have been either upregulated or expressed *de novo *post-HIV-infection. Proteins were uploaded and function-specific pathways were generated automatically for protein tyrosine kinases expressed in HIV-infected cells. Note that the newly discovered angiogenic pathways involve distinct protein tyrosine kinases and signaling proteins as described in the text. These pathways are *independent of *VEGFR2-VEGFC interactions as they do not interact with any of the proteins expressed in HIV-infected cells. Full names of all protein abbreviations and accession numbers are listed in Table 1.

Since ERBB2-PTK shuttles back and forth from the cell surface to the nucleus [73], the intracellular "PTK-pool" in HIV-infected cells is enhanced due to phosphorylation and activation of numerous additional kinases, regulatory enzymes, growth factors and other signaling proteins (Table 1, Figure 6 &7). The ERBB2 released in the circulation could therefore bind to cytokine-activated endothelial cells *in vivo *and induce cell proliferative signals, perhaps even before HIV has had a chance to replicate in these cells.

Expression of enhanced ERBB2 PTK activity has been associated with highly malignant (angiogenic) ovarian and breast cancers in women [74,75]. Activation of ERBB2-PTK- receptor in human umbilical vein endothelial cells *in vitro *stimulates proangiogenic factors *independent *of VEGF-signaling [76]. Studies in mouse cells have shown that upregulation of ERBB2 transcription induces angiogenic factors while suppressing antiangiogenic factors [77].

Among the numerous functions of the ERBB2 receptor, its involvement in the development of *fetal endothelium*[78] is most relevant to the present study since 90% of our HIV-induced proteins have been shown to be expressed during the growth, neovascularization/angiogenesis and development of the embryo. The ERBB2 receptor is activated by a wide range of pleiotropic growth factors and induces numerous signal transduction molecules which stimulate endothelial cell growth during the development of embryonic organs and angiogenesis [76,77]. A coordinated expression of ERBB2, with GRB2, PI3K, ZAP70 and FAK-tyrosine kinase and other signaling proteins in the experimentally HIV-infected cells is therefore anticipated to activate multiple PTK- regulatory pathways, inhibit apoptosis, enhance cell survival and stimulate endothelial cell growth *in vivo *(p = 3 × 10 – 2 × 10^-7^). These results indicate that predominant expression of ERBB2-PTK- activity triggered solely by HIV-replication, without any other intervention (infection or treatment), represents a new dimension of VEGF-*independent *pathways involved in neovascularization and angiogenesis (p = 4 × 10^-4^). Our data also suggest that biological processes of angiogenesis and embryonic development may be driven by common pathways.

##### Growth Factor Receptor-Bound Protein 2 (GRB2)

An important cell membrane-associated protein expressed in HIV-infected cells is the growth factor receptor-bound protein 2 (GRB2) which interacts with the *activated *ERBB2 receptor PTK. This protein is essential for the transduction of growth-promoting signals involved in morphogenesis as well as angiogenesis (Figures 6, 7) (p = 5 × 10^-8^).

GRB2 is associated with the activation of fetal genes through mitogen-activated protein kinase (MAPK) pathways and is central to the functionalities of PI3K and other growth-stimulating kinases [79] that are also upregulated by HIV-infection (Figure 4). Interaction of ERBB2 with the GRB2 protein is mediated by PI3K [66], while GRB2-associated scaffolding binding protein (GAB1) enhances capillary formation by coupling PI3K to VEGFR2 [80]. The coupling properties of PI3K and the binding of GRB2 to the activated ERBB2 in the presence of ZAP70-PTK and other kinases is highly significant as these interactions may not only stimulate endothelial cell growth along the angiogenic pathways but also influence cell migration and morphogenesis (p = 8 × 10^-12^), (Figures 6, 7).

##### Suppression of VEGF and its Cognate Receptor Tyrosine Kinase

The VEGF ligand and its cognate receptor VEGFR were *not *detected in the experimentally HIV-infected T-cells tested over a period of two years. Only a single acutely-infected culture showed basal levels of VEGF-C and its receptor VEGFR-2 *once *and was not reproducible in duplicate wells by MS. The absence in HIV-infected cells was completely unexpected since *t*he HIV-encoded Tat binds VEGFR via an arginine-glycine-aspartic-acid (RGD) region of homology and activates angiogenic pathways through the PTK activity of VEGFR [25,81]. However, the RGD domains are present in numerous integral plasma membrane proteins identified in this study including integrin and other cell adhesion proteins [82]. In addition, the binding of Tat to VEGFR is not as strong as the natural ligand (VEGF) and the angioproliferative processes are triggered *only *when Tat binds VEGFR in the presence of specific factors including IL-1 beta, TNF-alpha, IFN-gamma or other angiogenic cytokines [8,81,83-85].

As discussed above, our data has been corroborated by unrelated studies in which the expression of ZAP-70-PTK *suppresses *VEGF expression [44]. This fundamental knowledge has provided new insights into the tyrosine kinase-signaling pathways likely to be generated by numerous PTKs, serine threonine kinases and other signaling proteins identified in the present study. These mechanisms are similar to those reported for neovascularization in the development of embryos [75,86,87].

#### Step 4- Survival of Newly Formed Cells: Protein Kinase C and its Adapter Proteins

##### Protein Kinase C (PKC)

The HIV-infected cells expressed protein kinase C beta type (PKC, PKC-beta or KPCB), a serine/threonine kinase (Table 1; Figure 2). Activation of PKC augments upregulation of a series of tyrosine kinases, increases phosphorylation of proteins and leads to the production of numerous transcription factors [88] (p = 2 × 10^-5^). In the presence of MAPK, FAK2 and other kinases described herein, PKC may therefore play a significant role in preserving the cellular integrity during the development of a capillary network and other vascular processes *in vivo *[89-91]. Increased production of PKC in endothelial cells may also provide innate protection to these cells against complement-mediated injury during neovessel formation and possibly throughout the angiogenic growth [92].

An important functionality of PKC relevant to the present study is that upregulation of PKC-alpha/beta and MAPK in prostate and breast cancers, *downregulates *VEGF isomer D pathways and reduces tumor cell proliferation [93]. Downregulation of both VEGF and VEGFR in our HIV-infected cells could also be attributed to this unique property of PKC, as it stabilizes the overexpressed PTK activities while phosphorylating many proangiogenic protein substrates. Many PKC-beta2 *inhibitors *are therefore being tested for a more efficient inhibition of angiogenesis [94,95].

Our bioinformatics analyses indicate that the presence of PKC-beta is essential for maintaining an *activated state of major kinases *and other signaling proteins (C3, CRKL, ERBB2, ITGB5, MAPK3, PI3K, and PTK) that are concomitantly expressed in HIV-infected cells. This helps the proliferation of endothelial cells while protecting the HIV-infected cells from apoptosis. In addition, it stabilizes many critical biological processes necessary for angiogenesis (p = 2.6 × 10^-6^).

##### The Protein Kinase C-binding protein, NELL1

The expression of PKC was accompanied by the upregulation of two of its binding partners NELL1 and Annexin VI in HIV-infected T-cells (Table 1; Figure 2).

NELL1 is an extracellular matrix glycoprotein which belongs to a novel class of secreted polymorphic proteins that control mammalian cell growth and differentiation in the *presence *of PKC-beta [96]. The expression of this versatile protein is important because it contains multiple EGF-like repeat sequences, thrombospondin (TSP) N terminal sequence and five domains of von Willebrand factor (VWF), all of which are important for signal transduction and production of growth factors [97].

A significant finding of our proteomics studies is that NELL1 and VWF and several other upregulated proteins (ERBB2, CLR1/CELSR1 and LAMB2) and TSP-sequences (BAI1 and ADAMTS-9) contain EGF-like repeats. This phenomenon is critical for maintaining enhanced PTK activities and PKC-mediated stabilization of various upregulated cellular proteins essential for endothelial cell growth, differentiation and other vasculogenic functionalities (p = 2 × 10^-3^–2 × 10^-7^).

##### PKC-binding protein, Annexin VI (A6)

Another PKC-binding protein is annexin VI (A6), which was detected exclusively in HIV-infected cells (Table 1; Figure 2). Annexins are highly conserved plasma membrane proteins and many of its isoforms are involved in regulating Ca2+ efflux [98].

In two other proteomics-based studies different isoforms of annexins (A2, A6 and A11) were detected in HIV-infected cells, 12–42 hours post-HIV infection [99,100]. However, no PKC or PTKs were detected in these studies.

The co-expression of both A6 and PKC in our HIV-infected cells is important since the expression of MAPK in cells expressing A6 is PKC-dependent [101]. The upregulation of A6 in HIV-infected cells is therefore critical for the interaction of PKC with a number of binding partners [102]. These observations are consistent with our bioinformatics findings, indicating that PKC and its binding partners are vital for regulating the expression of other signaling proteins involved in multiple pathways (p = 2 × 10^-4^–2 × 10^-7^)

##### 14-3-3 protein gamma (143G)

The amount of PKC expression is regulated by a protein substrate designated 14-3-3 protein gamma (143G, also known as PKC inhibitor protein-1) [103,104]. This protein was downregulated 28% post-HIV infection compared to those present in the uninfected cells (Table 1; Figure 6). Our results were corroborated by another proteomics-based study in which 143G was downregulated 42-hours post-HIV-infection [29].

143G is an important signaling protein which regulates cytoskeletal architecture and mediates cellular effects of protein kinases (especially PKC) by binding specific peptide motifs of proteins that are phosphorylated on serine or threonine residues [105]. Since PKC is important kinase for the stability of protein-interactions and continued T-cell activation, downregulation of 143G may be essential for regulating and maintaining PKC-related signals in HIV-infected cells [104,106].

#### Step 5- Mitogenic Signaling Cascade: Mitogen-activated Protein Kinase

##### Mitogen-Activated Protein Kinase (MAPK3)

The mitogen-activated protein kinase (MAPK3, Syn: ERK1, MKO3), was induced *de novo *in HIV-infected cells (Table 1; Figure 2). This serine-threonine kinase is essential for numerous physiological and pathological functionalities including vascularization and mitogenesis [107,108]. MAPK can activate a large number of protein substrates by phosphorylation or dephosphorylation of proteins that are essential for the expression of cell cycle genes, new cell growth, proliferation of differentiated endothelial cells and stimulation of novel G-protein related signaling pathways [71,108].

Upregulation of MAPK3 in HIV-infected cells may have many consequences such as enhanced HIV-replication because it phosphorylates multiple HIV proteins (Tat, Rev, Nef and Gag) that regulate virus infectivity, reverse transcription, nuclear localization and packaging of the virus in infected cells [109-111]. The Gag-matrix protein is specifically utilized as one of the substrates for MAPK and Tat has been shown to activate MAPK pathways, which stimulate endothelial cell proliferation [112,113].

The expression of MAPK in HIV infected cells is mediated both by PKC-dependent and -independent pathways since MAPK and many other signaling enzymes and proteins identified in this study were upregulated synchronously and were stabilized by PKC (Table 1).

The MAPK signaling networks also involve PI3K/AKT pathways that are anti-apoptotic. Both of these kinases are expressed in HIV-infected cells (Table 1). In association with PI3K-signaling, MAPK regulates angiogenesis and promotes endothelial cell survival and sprouting [114]. Expression of these kinases is also critical for the cancer cells as well as for embryonic stem cell growth [86].

The MAPK3 signaling is important for promoting tumor vascularization *in vivo *[107]. When MAPK and other factors are released in the circulation *in vivo*, they bind to the cell surface of endothelial cells and activate them. Prolonged activation of endothelial cells by MAPK results in dysregulation of cell adhesion molecules that influence migration of the newly formed cells via changes in the cytoskeleton scaffolding [115,116]. These signals also stimulate smooth muscle proliferation and disrupt cadherin-mediated cell-cell interactions, which eventually promote microvessel formation and vascularization [101,115,117]. Taken together, our proteomics and bioinformatics analyses indicate that a well-synchronized expression of MAPK3, CRKL, ERBB2, PI3K, PKC, PTK and numerous adhesion molecules are involved in cell migration during neovascularization and angiogenesis (p = 3 × 10^-6^), (Figures 6, 7).

##### CRK-Like Adapter Protein (CRKL)

The CRK-Like adapter protein (CRKL) is essential for the activation of MAPK3 and it sustains phosphorylation of numerous proteins required for mitogenesis, cell proliferation, differentiation and migration (p = 5 × 10^-5^), [118-120]. This protein was expressed exclusively in HIV-infected cells (Table 1; Figure 2).

CRK is a member of an adapter protein family that binds to various tyrosine-phosphorylated proteins [121]. This protein has several Src-homology domains (SH2 and SH3) which recruit cytoplasmic proteins in the vicinity of tyrosine kinase through SH2-phosphotyrosine interaction. Thus, CRKL can bind to multiple sites of various signaling proteins and activate enzymatic cascades through their links to PI3K and other proteins [118,121].

In association with receptor protein tyrosine and GRB2-associated binder 1 protein, CRKL can form multimeric complexes with several growth promoting proteins involved in enhanced cell growth and invasion necessary for angiogenesis and metastasis [121,122].

Experiments in mouse embryo cells have shown that viral CRK is also essential for transducing signals for phosphorylating protein from extracellular matrix to focal adhesion targeting FAK another important kinases that was overexpressed in our HIV-infected cells [119](Figure 2). Thus, a coordinated expression of multiple tyrosine kinases and other enzymes (ERBB2, GRB2, CRKL, MAPK3, PKC, PI3K and FAK2) in HIV-infected cells may represent functional intermediates in triggering angiogenic pathways independent of VEGF activation (Figures 6, 7).

#### Step 6- Balanced Cell Growth: "Anti-angiogenic" G-Protein Coupled Receptors

##### Brain-Specific Angiogenesis Inhibitors 1 and 3

Two cellular proteins, the brain-specific angiogenesis inhibitors -1 and -3 (BAI1 and BAI3 respectively) were slightly upregulated in HIV-infected cells (Table 1; Figure 2). Both BAI1 and BAI3 are adhesion-type guanine nucleotide-binding (G) protein coupled receptors (GPCRs) essential for mediating receptor tyrosine kinase (PTK) and GTPase-associated signaling pathways [123,124]. A major function of these cell-surface receptors is to protect the tissue from increased vascularization by regulating the expression of excessive proangiogenic factors induced by various insults such as hypoxia, ischemia, inflammation or tumorigenesis (p = 7 × 10^-7^), [125-127].

The roles of BAI1 and BAI3 in HIV-infected human cells are not clear. However, in the human brain, BAI1 is a p53- target gene important for signal transduction [128,129]. Our bioinformatics analyses suggest that these GPCRs may be similar to other "embryonic" proteins that have been dysregulated by HIV-infection and may be necessary to sustain different PTK-mediated cellular processes involved in cell-adhesion and protein-protein interactions necessary for enhanced virus replication, cell growth, migration and invasion. Expression of BAI1 and BAI3 receptors in HIV-infected T-cells also suggests that both proangiogenic and anti-angiogenic signals are necessary for maintaining a balance of tyrosine kinase phosphorylation and focal adhesion signaling to restrict pathologic angiogenesis [125,126,129]. The BAI1 protein may also mediate signals for enhanced cell invasion and migration because it contains thrombospondin-type repeats [130].

#### Step 7- Cell Adhesion, Differentiation & Migration: Focal Adhesion Kinase & Receptors

##### Focal Adhesion Tyrosine Kinase (FAK2)

Of all the kinases and enzymes identified in our experimentally infected cells, the focal adhesion tyrosine kinase 2 beta (FAK2: synonyms Pyk2/RAFTK/CAK beta) displayed the highest quantities (Table 1; Figure 2).

Activation of FAK2 and regulation of cell adhesion are associated with changes in cytoskeletal signaling primarily due to its interaction with growth factor receptors and integrins [131]. Both of these classes of proteins were also upregulated post-HIV-infection (Figure 7). FAK2 is a calcium-dependent tyrosine kinase activated in response to calcium flux and it regulates Ca2+-induced ion channels through phosphorylation [132,133]. The catalytic activity of FAK2 promotes downstream activation of many kinases including MAPK3 and signaling proteins along novel pathway [133]. These interactions have been associated with angiogenesis among other pathological conditions [2,134].

In HIV-infected cells, Tat protein may enhance focal tyrosine phosphorylation which induces signals for cytoskeletal reorganization in endothelial cells [135,136]. In human brain endothelial cells FAK2 is considered essential for cell migration and permeability of the microvasculature [133,136].

Cell adhesion is particularly critical for the newly synthesized endothelial cells to adhere together *in vivo *as they tend to differentiate into functional entities [2,91]. Thus, FAK2 plays a vital role in endothelial cell growth, proliferation, survival, motility, migration and differentiation (p = 2 × 10^-4^), [119,137,138].

Expression of adhesion molecules is also essential for angiogenesis in the embryo (p = 4 × 10 – 2 × 10^-7^).

The numerous diffusible factors described in this study provide compelling evidence that binding of several members of adhesion molecules to their cognate receptors on the endothelial cells *in vivo *would be expected to promote FAK2 tyrosine kinase-coordinated signals for endothelial cell proliferation, adhesion, morphogenesis and angiogenesis [119,120,134]. Our bioinformatics and statistical analysis indicates that the FAK2- PTK activity alone is critical for angiogenic processes (p = 2.6 × 10^-3^). A well-coordinated expression FAK2 with other protein tyrosine kinases (ZAP70, ERBB2, ITB5), and many adapter/signaling proteins in HIV-infected cells is highly significant for angiogenesis (p = 1.3 × 10^-5^).

##### Integrin alpha-v- beta-5 (ITB5) and Fibronectin (FINC)

Both integrin alpha-v-beta-5 (ITB5) and fibronectin (FINC) were upregulated in HIV-infected cells but ITB5 was not detected in the uninfected control cells (Table 1). Integrins are a family of adhesion receptors present in the extracellular matrix while FINC is an important factor that binds to integrins as well as to many other cell surfaces proteins involved in cell adhesion and motility [131].

A large number of proteins bind to integrins via the RGD as well as the non-RGD domains [82,139]. The MAPK cooperates with integrin alpha5 beta1 to enhance migration of endothelial cells and promote neovessel formation during vasculogenesis and angiogenesis [140,141].

Although in HIV-infected cells RGD motifs present in the Tat bind to VEGFR in primary Kaposi's sarcoma and other endothelial cells, these domains are not specific to Tat as they are present in numerous cell surface receptors and cell adhesion molecules[82]. Interactions between fibronectin, integrin and other cell surface molecules also enhance production of angiogenic factors involved in wound healing, repair of blood vessels, development of embryonic tissues and maintenance of cell shape [131,140,142].

The development of embryonic organ systems also depend on integrins that are required for the differentiation of the visceral endoderm [142,143]. Activation of these multifunctional proteins is essential for diverse cellular functions, including cell-cell interactions, cell adhesion, cell aggregation, cell migration, cell cycle progression, differentiation, inflammation, angiogenesis, and maintenance of homeostasis in most animal species[144,145] (p = 1.6 × 10 – 9 × 10^-8^).

The integrin was synchronously upregulated in HIV-infected cells with numerous cell-surface signaling proteins such as ERBB2, PI3K discussed earlier. These findings are in agreement with the report that PI3K signaling pathways are initiated by ERBB which upregulates beta1-integrin functions [146]. Thus, the overexpression of ERBB-PTK, GRB2, ZAP-70, MAPK, dysregulation of integrins and upregulation of adhesion kinase, all contribute to the formation of vasculature and promote angiogenesis via novel VEGF-independent pathways (Table 1) [82,139].

##### Expression of Nitric-oxide Synthase (NOS) and Downregulation of PPAC

A critical enzyme expressed in our experimentally infected cells was the nitric oxide synthase (NOS or NS2A) (Figure 2). This enzyme is located in the plasma membrane and transported to the cytoplasm to regulate multiple functions [147]. NOS is activated in response to cellular stress and it regulates vascular functions including endothelial cell migration necessary for angiogenesis [147].

Expression of NOS in HIV-infected cells is considered to be important as it also *inactivates *the low molecular weight phosphotyrosine protein phosphatase (PPAC, Syn. HCPTPA), an enzyme that *impairs *the VEGF-mediated *autophosphorylation *[36,148]. Although PPAC phosphatase was detected in the uninfected T-cells, its expression was completely downregulated (shut-off) after HIV-infection (Table 1; Figure 6). PPAC is an important regulator of VEGF-mediated signaling and it has been shown to prevents endothelial signaling downstream of VEGFR, which *inhibits *angiogenic responses, cell proliferation and migration [36]. Since both VEGF and VEGFR-PTK were not expressed in HIV-infected cells, the *absence *of PPAC would be essential for maintaining *phosphorylation *of various other tyrosine kinases and activating endothelial cell growth *in vivo *(p = 3 × 10^-7^).

The upregulation of NOS in combination with a well-coordinated expression of multiple PTK- proteins (ERBB2, ZAP70, FAK, GRB2, CRKL), serine-threonine kinases and other signaling proteins in the absence of PPAC, would therefore enhance phosphorylation of substrate proteins and maintain a downregulated state of VEGFR kinase in HIV-infected T-cells through VEGF-independent pathways (p = 2 × 10^-4^) (Figure 5).

#### Step 8- Morphogenesis and Cell Migration: Laminins and Cell Adhesion Molecules

##### Laminins

Many different types of laminins (alpha, beta and gamma chains) were expressed in our experimentally HIV-infected T-cells but the quantity of laminin beta-2 chain (LAMB2) precursor was significantly higher than other laminins (Table 1; Figure 2). About the same quantity of laminin alpha-5 chain (LAMA5) was expressed in both the HIV-infected and uninfected control cells, and only LAMB2 was upregulated in HIV-infected cells. Laminin beta-3 chain (LAMB3 precursor) and laminin gamma-1 chain (LAMC1) were detected only once at low levels and therefore were not included in the analyses.

Laminins are a family of morphogenic glycoproteins, which are secreted and incorporated into the extracellular matrices of many tissues. These proteins bind to different isoforms of integrins and other cell surface receptors to form cellular structural scaffoldings [149,150].

Thus, LAMB2, which is present in the basement membranes of many tissues, is essential for cell proliferation, migration and differentiation of cells in early development of embryos [149]. This protein has EGF-like extracellular domains crucial for rolling up and adhesion of endothelial cells to form microvessels [151]. Statistical analysis shows that the coexpression of LAMB2, MAPK3, CRKL, FAK2, with ERBB2, GRB2, INC, NOS2 TNR9, MYLK, PKC, TP53BP1 and numerous PTK signaling proteins is highly significant for the survival, morphogenesis, migration and microvessel formation of cells (p = 6 × 10^-7^) [25,131,152-154].

##### Cadherin EGF LAG Seven-Pass G-Type Receptor 1 (CLR1/CELSR1)

Among the membrane-bound proteins that were upregulated in HIV infected T-cells, cadherin EGF LAG seven-pass G- coupled protein receptor (GPCR) type 1 (CELSR1,syn.CLR1) was detected frequently in HIV-infected cells although the expression levels of this protein were not increased significantly compared to the uninfected cells. The HIV-VPR protein has been shown to modulate higher expression of cadherin and integrins alpha5 and alpha6 in T-cells. This interaction not only enhances cell survival but also increases virus spread and modulate expression of many cell surface molecules [155]. As discussed previously, expression and prolonged activation of MAPK3 in HIV-infected cells results in disruption of cadherin-mediated cell-cell interactions, which increases cell migration, a function highly relevant to angiogenesis [115].

Cadherins are considered as lineage-specific differentiation markers for endothelial cell. The polymorphic EGF-like extracellular domains of these proteins interact with catenin and other signaling proteins and activate enzymes, ion channels, a process that facilitates cell adhesion and migration [123,124,156].

These proteins are expressed at peak levels during perinatal vascular development and are involved in morphogenesis particularly in connecting similar cell types in a homophilic manner [157].

During embryonic development, cadherin is linked to microfilament and cytoskeletal proteins which cooperatively influence cell adhesion and tubular morphogenesis (p = 3 × 10^4^).

##### Protocadherin Focal Adhesion Targeting type 2 (FAT2) Protein

The protocadherin **f**ocal **a**dhesion **t**argeting (FAT) protein type 2 belongs to a novel superfamily of membrane associated cadherins. FAT2 was expressed exclusively in HIV-infected cells (Table 1) and is homologous to Drosophila FAT proteins (FAT1, FAT2, FAT3 and FAT4) [158,159].

Expression of FAT2 is essential for cell recognition, regulation of polarity during cell adhesion, microvessel formation and correct morphogenesis of the embryo [159,160]. Protocadherins also regulate angiogenesis in specific brain regions or a subset of blood vessels in the developing vertebrate brain [157,158]. However, expression of FAT2 mRNA in adults is associated with numerous cancers such as highly metastatic/angiogenic ovarian and head and neck cancers [158].

##### Golgi apparatus Protein 1 (GLG1)

The Golgi apparatus protein 1 (GLG1) was expressed exclusively in HIV-infected cells (Table 1; Figure 2). GLGI, also known as E-selectin-type integral membrane protein, Golgi sialoglycoprotein (MG-160), E-selectin ligand 1 (ESL-1) or cysteine-rich fibroblast growth factor (FGF) receptor CFR-1, is normally expressed on endothelial cells and mediates morphogenesis and trafficking of cells through the vascular endothelium (p = 2 × 10^-5^), [161]. The expression of GLG1 is enhanced on lymphocytes that are in contact with the endothelium, because it interacts with adhesion molecules and their cognate receptors present on the endothelial cell [162].

#### Step 9- Cell Permeability & Sprouting: Myosin Light Chain Kinase & Aggrecans

##### Myosin Light Chain Kinase Smooth Muscle/Non-muscle Isozyme (MYLK)

The myosin light chain kinase smooth muscle/non-muscle isozyme (MYLK or KMLS), was upregulated in T-cells after HIV infection (Table 1; Figure 2). MYLK is an important cytoplasmic kinase expressed in many different cell types including neurons, glia, and endothelial cells [163,164]. Expression of this enzyme is vital for phosphorylation of cellular proteins involved in contraction of cells, regulation of cell shape and formation of new structures such as gap junction, tubular morphogenesis and cell permeability, all critical steps before cell migration toward a chemotactic gradient [163,164].

Bioinformatics analyses of HIV demonstrated that synchronous expression of MYLK in our experimentally infected T-cells with numerous cell adhesion molecules, laminins and extracellular matrix proteins, kinases and other enzymes (C3, ERBB2, FINC, MYLK, NOS2A, PI3K, PKC, FAK2) is highly significant for microvessel formation and migration of newly formed cells (p = 2 × 10^-3 ^to 2.6 × 10^-6^).

##### A Disintegrin And Metalloproteinase with Thrombospondin Type I Sequence

**ADAMTS-9 **(**A D**isintegrin **A**nd **M**etalloproteinase with **T**hrombo**Sp**ondin (TSP)-Type I sequence motifs), contain an ADAM protease domain [165] as well as **t**hrombo**s**pondin 1 repeats [166,167]. This protein was expressed in HIV-infected T-lymphocytes (Table 1).

Morphogenesis of cellular structures requires well-controlled proteolytic activities that are regulated by proteinases. ADAMTS are specific metalloproteases or aggrecanase localized in the extracellular space critical of the cleavage of large *aggregating *proteoglycans or aggrecans normally expressed in *growing tissues *[167,168]. Compared to other aggrecanase, ADAMTS-9 is more responsive to proinflammatory cytokines, such as TNF and chemokines expressed in HIV infected cells *in vitro *or *in vivo *[169].

An altered expression of ADAMTS enzyme contributes to the permeability and migration of cells from tissues, a feature essential for microvessel formation [167,170]. ADAMTS- 9 can punctuate basement membranes of the endothelial cells in front of the sprouting vessel such that the proliferating cells can penetrate existing vessels through the small microscopic perforations [166].

The TSP-containing proteins were initially reported to exhibit anti-angiogenic and tumor suppressor activities in mice [171], ADAMTS- matrix metalloproteinases with thrombospondin repeats have since been considered important factors for angiogenesis and other endothelial cell functions [172]. Thus, co-expression of ADAMTS9, C3, FN1, MAPK3, PKC, TNFR9 and TP53BP1 in the presence of ERBB2, LAMB2 and other proteins in the experimentally infected cells is significantly associated with numerous biological processes in angiogenesis p = 2 × 10^-3^).

##### Complement Receptor 3 (CO3/C3 Peptidase)

As previously discussed, the complement receptor 3 (CO3/C3) is one of the first responders of the innate immunity. This protein was expressed exclusively in HIV-infected T-cells (Table 1; Figure 2). In addition to its involvement in HIV-infection and pathogenesis, the C3 protein is also associated with chemotaxis, muscle contraction and enhanced permeability of small blood vessels [55,56,59]. C3 plays a significant role in protecting endothelial cells and HIV-infected T-cells from apoptosis during virus replication. Furthermore, C3 also regulates complement activation during angiogenesis via PKC-dependent and PKC-independent pathways [92].

Expression of C3 peptidase in the extracellular matrix has been shown to increase restoration of morphologically intact myofibers and enhanced permeability of vessels after trauma-induced vascular disruption [173]. Herein we show through bioinformatics analyses that concomitant expression of the C3 complement regulatory system in the presence of FINC, LAMB2, MYLK, PKC, FAK2, PI3K, ERBB2, MAPK3, ITG5, and other proteins is critical for increased production of chemotactic and proangiogenic factors [59,92,174], (p = 2.6 × 10^-6^).

#### Step 10- Preservation of Differentiated Endothelial Cells: Von Willebrand Factor

##### Von Willebrand Factor (VWF)

The Von Willebrand factor (VWF) binds to platelet receptors and activates these cells [175]. The VWF- precursor was upregulated in the experimentally HIV-infected T-cells, compared to the uninfected counterpart cells (Table 1). This factor is normally produced by endothelial cells and secreted in the plasma. Diverse physiological functions performed by VWF include cell adhesion, cell migration, cell cycle progression and differentiation of endothelial cells [175-178]. The VWF also acts as a permeability barrier for endothelial cells and is vital for the transport of the coagulation factor VIII in the plasma [178].

**Figure 8:**
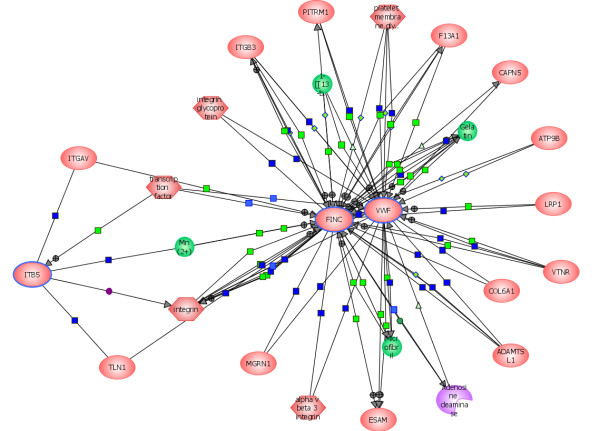
**Proteins Involved in Preservation of Differentiated Endothelial Cell Phenotypes**. Protein-interaction pathways responsible for maintaining differentiated state of endothelial cells. Full names of all protein abbreviations and accession numbers are listed in Table 1.

While an increased expression of VWF has been linked directly or indirectly to HIV infection of *endothelial *cells [179], it also augments activation and adhesion of aggregated platelets and interacts with integrins and FINC in order to maintain cellular integrity (Figure 8) [180]. Enhanced production of VWF is also indicative of vascular injury, thrombus formation, inflammation and angiogenesis [176,177]. In HIV-infected individuals an increase in the plasma levels of VWF is considered a marker of endothelial cell proliferation resulting in abnormal patterns of angiogenesis [181]. Patients with highly dysplastic anal warts, cervical and vulvar cancers also show statistically significant correlations with the upregulation of VWF and enhanced capillary formation, microvessel density and angiogenesis [22].

One of the final steps in the numerous complex processes involved in angiogenesis is the maintenance of cell adhesion while the newly formed endothelial cells are being differentiated *in vivo*. The VWF modulates these processes and sustains the differentiated state of these cells (Figure 8). In addition, the blood flow during the development of a network of new blood vessels is also facilitated by VWF. Thus, this soluble factor provides numerous functions, particularly in the presence of numerous coordinately expressed proteins such as ITGB5, PKC, C3, F1NC, MAPK3, ERBB2, GRB2, FAK2, ZAP70 and numerous adhesion molecules during HIV-infection (p = 9.1 × 10 – 8 × 10^-7^).

## Conclusion

1. We have provided the first direct evidence that chronic HIV-replication in T-cells, without any treatment or co-infection with another pathogen, produces angiogenic or proangiogenic proteins. 88% proteins are localized in the plasma membrane and extracellular matrix, while more than 90% of the upregulated proteins are similar to those expressed during wound healing, regeneration and embryonic neovascularization or angiogenesis (p = 10^-4 ^to 10^-12^).

2. Based on the protein-protein interaction pathway analyses, we have identified key events during angiogenesis and proposed comprehensive putative mechanisms by which a well-coordinated expression of several families of proteins (cell surface receptors, kinases, regulatory enzymes, growth factors, adhesion molecules and other signaling proteins) can generate a network of interactions along multiple novel pathways leading to T-cell activation, transcriptional and translational reprogramming, cell cycle changes, cell proliferation, cell growth, migration, cell adhesion, sprouting, microvessel formation and maintenance of differentiated endothelial cells that are highly significant for neovascularization or angiogenic responses (p = 1.0 × 10^-11^).

3. While the *in vitro *results cannot be correlated directly to the consequences of HIV-infection *in vivo*, a unique finding of our bioinformatics analyses is that activation of T-cells results in the production of a diverse array of protein tyrosine kinases (PTKs), serine-threonine kinases, lipid kinases, adhesion molecules and other diffusible signaling proteins. The abundance of multiple PTKs and other kinases initiates novel angiogenic pathways *independent *of VEGF-signaling while *suppressing *activation of VEGFR-PTK activity. This mechanism is similar to that observed in neovascularization in the developing embryo.

4. Since T-cells and monocytes/macrophages are the primary cell types to be infected at the portal of entry *in vivo*, the HIV-infected T-cells may induce ERBB2 and other PTK-related pathways soon after infection and VEGF-independent pathways may possibly precede HIV-infection of endothelial cells. It is possible however, that in chronically HIV-infected individuals, both VEGF-dependent and VEGF – independent pathways may be operative as many different cell types are infected by HIV and other pathogenic viruses and microorganisms. Dominance of one or both pathways would depend on the individual's genetic predispositions, co-infections with other pathogenic organisms and environmental factors that affect the disease outcome. The knowledge that HIV-infection alone can induce synthesis of multiple proangiogenic signals *independent *of VEGFR-stimulus adds a new dimension to our understanding of HIV-induced vasculopathies and for identifying clinically relevant angiogenic markers by gene silencing and translational studies *in vivo*.

## Competing interests

The authors declare that they have no competing interests.

## Authors' contributions

SR conceived, designed and performed proteomics experiments with the technical help from Zisu Mao for cell culture and two-dimensional gel electrophoresis; BL helped in identification of proteins by mass spectrometry (MS) and supervised Jane M.C. Chan in MS; SR, JSY and AL performed literature searches and analyzed the data. All authors except Bruce Lai (unavailable) read and approved the final manuscript.

SR contributed reagents/materials/analysis tools.

## Author's information

Suraiya Rasheed is a Professor of Pathology and Director, Laboratory of Viral Oncology and Proteomics Research at the Keck School of Medicine, University of Southern California, Los Angeles. She has expertise in molecular biology of HIV and proteomics research. Her laboratory discovered the first *ras *oncogene in the form of the Rasheed Rat Sarcoma virus and the Feline Gardner Rasheed (*Fgr*) oncogene in a feline sarcoma virus. This laboratory has also isolated a novel HIV strain (HIV-Ibng) from Nigeria, a unique cat endogenous retrovirus (RD114) and the naturally occurring amphotropic murine leukemia viruses that replicate in human cells. These retroviruses are used globally for constructing vectors for gene transfer.

Bruce Lai is a computer scientist and is an expert in mass spectrometry; Jasper Yan and Adil Hussain are students.
